# Liver Cirrhosis With Encapsulating Peritoneal Sclerosis: A Case Report

**DOI:** 10.7759/cureus.100195

**Published:** 2025-12-27

**Authors:** Emery Boudreau, Rosemarie Matte, Christian Wilke

**Affiliations:** 1 Surgery, Dartmouth Hitchcock Medical Center, Lebanon, USA; 2 Pathology, Concord Hospital, Concord, USA; 3 Surgery, Concord Hospital, Concord, USA

**Keywords:** abdominal cocoon, alcohol related cirrhosis, case report, peritoneal sclerosis, sclerosing encapsulating peritonitis

## Abstract

A 72-year-old male with a history of alcohol use disorder complicated by cirrhosis and chronic thrombocytopenia, hypertension, chronic back pain, a congenital solitary kidney, and active tobacco use presented with a one-year history of intermittent right upper quadrant pain. Preoperative evaluation suggested symptomatic cholelithiasis, and he was brought to the operating room for laparoscopic cholecystectomy. Intraoperatively, dense, fibrotic tissue approximately 1-2 cm thick was found encasing the liver, with similar deposits over the antimesenteric surface of the colon, scattered stippling across the peritoneum, and a 1 cm nodule attached to the anterior abdominal wall peritoneum in the right upper quadrant. Given these unexpected findings, the cholecystectomy was deferred, and biopsies were obtained. Histopathological analysis revealed peritoneal sclerosis. Encapsulating peritoneal sclerosis is a rare condition most commonly seen in small-bowel involvement among post-transplant patients following peritoneal dialysis. We report this unusual case of advanced sclerosing peritonitis primarily affecting the hepatic surface in a patient without any history of peritoneal dialysis or transplantation. This unique presentation underscores the importance of further investigation into the pathophysiology and clinical spectrum of this rare entity.

## Introduction

Sclerosing encapsulating peritonitis (SEP) is a rare syndrome characterized by chronic inflammation of the peritoneum. The specific etiology is not known, and the primary condition is thought to be idiopathic [1-6]. It has also been described as a secondary outcome of conditions or treatments that cause peritoneal inflammation, such as peritoneal dialysis (PD), peritovenous or ventriculoperitoneal shunts, intraperitoneal chemotherapy, liver transplantation, cirrhosis, gastrointestinal malignancy, endometriosis, sarcoidosis, or abdominal tuberculosis, and tends to be a chronic process [1-7]. A review done in 2016 identified 118 cases of SEP in the literature. The majority of these patients presented with abdominal pain, nausea, and weight loss. Many had episodic bowel obstructions and intraoperatively were found to have encasement of primarily small bowel [1]. This process has also been described as an abdominal cocoon. In the limited available literature, it does tend to affect the small bowel most frequently, often in patients with a diagnosis of cirrhosis or end-stage renal disease on PD [1-4,7]. Until this report, there have been no described cases where the liver was the primary location of encapsulation, or to the degree that we saw in this case.

## Case presentation

**Figure 1:**
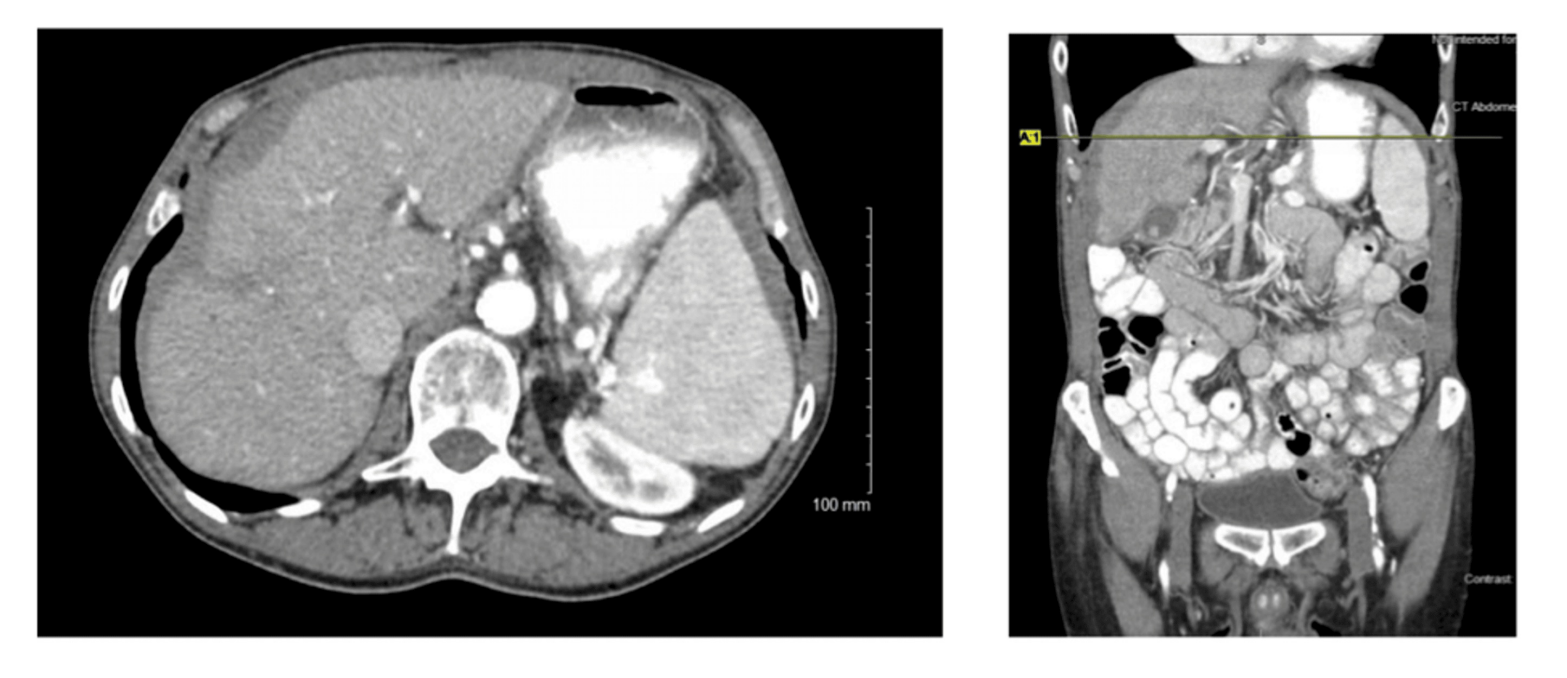
Preoperative CT images Fibrotic-appearing liver and perihepatic hypoattenuating substance interpreted as ascites.

This patient has a past medical history of alcohol use disorder (abstinent for four months), resultant cirrhosis and chronic thrombocytopenia, hypertension, chronic back pain, congenital solitary kidney, active tobacco use, and long-standing post-prandial right upper quadrant (RUQ) pain. He presented with one to two episodes of pain per day following meals, starting roughly one year prior to evaluation. Denied any additional systemic symptoms, no jaundice or acholic stools. No changes in bowel habits or urination. He had noted a 10 lb unintentional weight loss in the past six months. An abdominal ultrasound was obtained in the workup of his RUQ pain, which showed cholelithiasis, cirrhotic-appearing liver, and common bile duct dilation to 9.2 mm. An abdominal CT demonstrated a small and nodular-appearing liver consistent with cirrhosis, retroperitoneal and esophageal varices, gallstones, and what was initially interpreted as perihepatic ascites (Figure 1). Preoperative lab data included aspartate aminotransferase (AST) 36 U/L, alanine aminotransferase (ALT) 38 U/L, alkaline phosphatase (ALKP) 54 U/L, total bilirubin 1.0 mg/dL, lipase 33 U/L, and platelets 106 K/uL. He was then referred to general surgery at our community hospital. Evaluation confirmed hepatomegaly and mild RUQ tenderness on exam. He was counseled extensively on the risks of laparoscopic, possibly open cholecystectomy, with increased risks given his liver disease. After preoperative evaluation with his PCP and cardiology, the patient did wish to proceed with surgery due to the ongoing symptoms.

**Figure 2:**
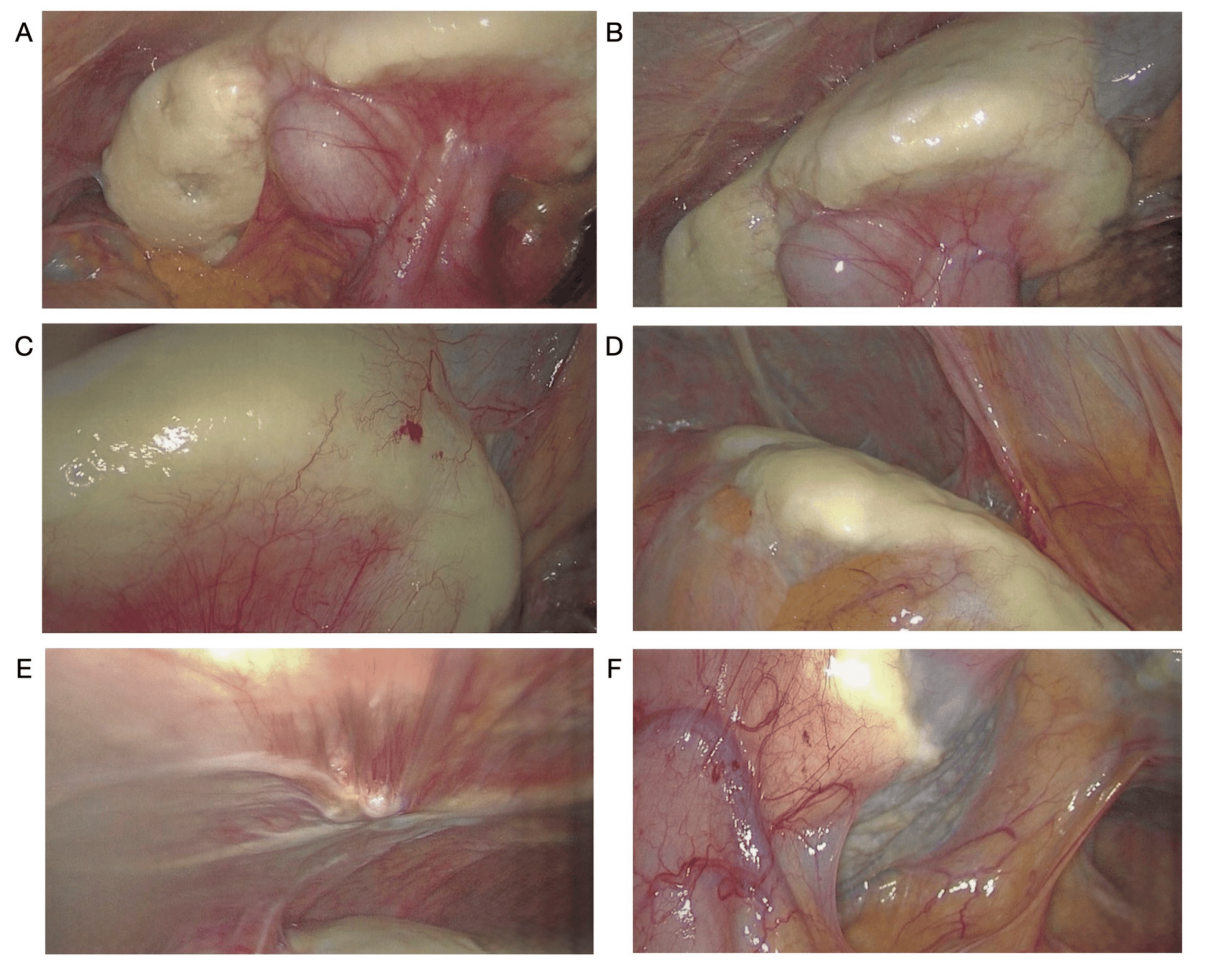
Intraoperative images A, B: Encasement of anterior hepatic surfaces and gallbladder with tenting of the duodenum. C: Left hepatic lobe with neovascularization. D: Deposits over antimesenteric descending colon. E: Peritoneal inserts to the anterior abdominal wall. F: Thinner encasement over caudate lobe.

The patient was taken to the operating room for a planned laparoscopic, possibly open cholecystectomy. The abdomen was accessed under direct visualization using the Hasson open technique in the supraumbilical position. After insufflation, the abdomen was inspected, and we observed dense tissue encasement around the liver, roughly 1-2 cm thick. There were additional deposits over the antimesenteric colonic surface, stippling to the peritoneum, and a 1 cm nodule implanted on the anterior abdominal wall. Interestingly, there were no signs of acute or chronic cholecystitis. The duodenum was adherent to the medial aspect of the gallbladder, tented up from the peritoneal process (Figure 2). Excisional biopsies of the left liver lobe and peritoneal nodules were taken; the deposits were hard and avascular. Intraoperative frozen sections noted “hypocellular hyalinized tissue.” Given these unexpected findings, we did not proceed with elective cholecystectomy at that time. The procedure was aborted to allow for additional workup and final pathology results.

The patient was admitted to the hospital overnight for observation. He did very well postoperatively, tolerated a diet with ongoing post-prandial discomfort but no escalation of symptoms. He was discharged home on postoperative day one. Infectious disease was consulted, and they felt his history and presentation did not suggest an infectious etiology for the intraoperative findings. Their recommendations included the addition of fungal and bacterial studies, AFB stains, and cultures from the intraoperative specimens. All of these tests were negative.

**Figure 3:**
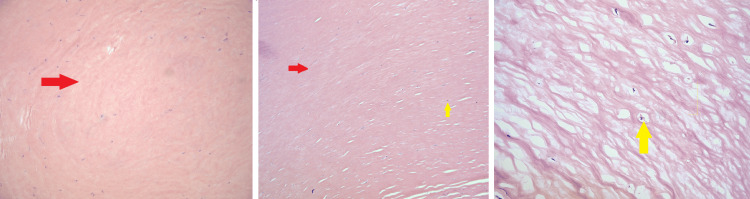
Biopsy histologic slides Dense paucicellular hyalinized tissue without cytologic atypia (red arrows), focal chronic inflammatory cells (yellow arrows).

The final pathologic examination showed thick, dense, and paucicellular hyalinized tissue with a denuded mesothelial lining. Focal chronic inflammatory cells were present, comprised primarily of lymphocytes. No granulomata, cytologic atypia, or overt malignancy was identified. Smooth muscle actin (SMA) and desmin immunohistochemical stains were negative, signifying that no smooth muscle differentiation was present in the tissue. A trichrome stain was positive, supporting the fibrotic nature of the tissue. In addition, AFB and GMS stains were negative for acid-fast bacilli and fungal organisms, respectively. A Congo red stain was negative for amyloid deposition. The overall findings were suggestive of a sclerosing peritoneal process. The pathology slides were also reviewed at an outside institution, given the unusual clinical presentation of this patient. They were in agreement that these results were most consistent with a diagnosis of peritoneal sclerosis (Figure 3).

Our patient was advised to continue with a low-fat diet as we were unable to safely proceed with a cholecystectomy at the time of surgery. His symptoms of RUQ post-prandial pain in the presence of gallstones are still suggestive of symptomatic cholelithiasis as a diagnosis, but with the tenting of the duodenum encased in this fibrosing process, we cannot rule out peritoneal sclerosis as a contributing factor to his symptoms. He was referred to a multidisciplinary liver team, along with a transplant surgeon, for continuation of his workup and any further testing, such as a hepatobiliary iminodiacetic acid (HIDA) scan or upper GI study. It would have been very difficult to obtain a critical view of safety, given the degree of sclerosis surrounding the gallbladder infundibulum. For this reason, we recommended he pursue surgery with a transplant surgeon or hepatobiliary specialist if he did ultimately undergo cholecystectomy. So far, the patient has declined to see a specialist. At his most recent primary care visit one year after surgery, he is overall doing well. His weight is stable, and there are no signs of jaundice. He endorses occasional RUQ pain, which is well managed with dietary changes.

## Discussion

In our review of the literature, there were no described cases where the liver was the primary location of encapsulation, or where hepatic encapsulation was noted to the degree we observed in our patient, as demonstrated in the images above [1-7]. While our patient did have a diagnosis of cirrhosis, his renal function was normal, and he’s never required PD. He had undergone multiple paracentesis for ascites when he was heavily drinking, with the last procedure back in 2005, over 20 years prior to his surgery and onset of symptoms. His only known definitive risk factor for SEP is a diagnosis of cirrhosis.

Most cases of SEP are diagnosed at the time of operative intervention for bowel obstruction, and symptoms often improve after exploration with adhesiolysis and membrane resection [1,5]. SEP can also be treated conservatively in some patients with bowel rest followed by slow diet progression. There are several medical therapies described, including steroids, colchicine, and tamoxifen, which reduce collagen synthesis, provide anti-inflammatory actions, or inhibit fibroblastic production of transforming growth factor, respectively [1,5-7]. Our patient’s case is a unique example of this occult disease process. It required us to reassess the risks and benefits of proceeding with surgery once identified, and we ultimately referred him to a team that would have experience with medical or operative interventions for this disease process. While still rare in any practice, transplant specialists are often involved in the management of SEP given its association with PD.

## Conclusions

Encapsulating peritoneal sclerosis is a rare but described process that most often affects the small bowel and typically presents in transplant patients after PD. This diagnosis requires a high degree of clinical suspicion and often is not recognized until a patient is in the operating room. When faced with unexpected findings intraoperatively, a surgeon must reevaluate the risks and benefits to that patient of proceeding with surgery. In some cases, it is most appropriate to abort or defer a planned procedure until more information can be gathered, and these findings and next steps can be discussed with the patient. We present a case of advanced sclerosis primarily affecting the hepatic surface in a patient without a history of PD or transplantation. This is a unique presentation of this rare process, highlighting the need for continued investigation into this pathology.
